# HMGB1 promotes CXCL12‐dependent egress of murine B cells from Peyer's patches in homeostasis

**DOI:** 10.1002/eji.202049120

**Published:** 2021-06-16

**Authors:** Lorenzo Spagnuolo, Viola Puddinu, Noémie Boss, Thibaud Spinetti, Anne Oberson, Jerome Widmer, Inès Mottas, Christian Hotz, Marco E. Bianchi, Mariagrazia Uguccioni, Carole Bourquin

**Affiliations:** ^1^ Chair of Pharmacology Department of Medicine Faculty of Science University of Fribourg Fribourg Switzerland; ^2^ School of Pharmaceutical Sciences University of Geneva Geneva Switzerland; ^3^ Institute of Pharmaceutical Sciences of Western Switzerland University of Geneva Geneva Switzerland; ^4^ Department of Anesthesiology Pharmacology, Intensive Care and Emergency Medicine University of Geneva Geneva Switzerland; ^5^ Department of Intensive Care Medicine, Inselspital Bern University Hospital University of Bern Bern Switzerland; ^6^ Division of Genetics and Cell Biology San Raffaele University and Scientific Institute Milan Italy; ^7^ Institute for Research in Biomedicine Universitá della Svizzera italiana Bellinzona Switzerland; ^8^ Department of Biomedical Sciences Humanitas University Milan Italy

**Keywords:** B‐cell migration, Gut immune regulation, Peyer's patches, IgA, HMGB1

## Abstract

High mobility group box‐1 protein (HMGB1) is an alarmin that, once released, promotes inflammatory responses, alone and as a complex with the chemokine CXCL12. Here, we report that the HMGB1–CXCL12 complex plays an essential role also in homeostasis by controlling the migration of B lymphocytes. We show that extracellular HMGB1 is critical for the CXCL12‐dependent egress of B cells from the Peyer's patches (PP). This promigratory function of the complex was restricted to the PPs, since HMGB1 was not required for B‐cell migratory processes in other locations. Accordingly, we detected higher constitutive levels of the HMGB1–CXCL12 complex in PPs than in other lymphoid organs. HMGB1–CXCL12 in vivo inhibition was associated with a reduced basal IgA production in the gut. Collectively, our results demonstrate a role for the HMGB1–CXCL12 complex in orchestrating B‐cell trafficking in homeostasis, and provide a novel target to control lymphocyte migration in mucosal immunity.

## Graphical abstract

Through a set of *in vitro* and *in vivo* experiments in mice, we show that HMGB1, in complex with the chemokine CXCL12, controls homeostatic B‐cell egress from Peyer's patches, and that inhibition of the HMGB1‐CXCL12 complex by glycyrrhizin decreases intestinal IgA production. These observations uncover an important role in homeostasis for extracellular HMGB1, to date considered only as a pro‐inflammatory molecule.

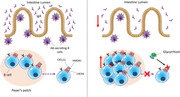

## Introduction

A key property of immune cells is their high mobility, which allows them to circulate throughout the body, colonize lymphoid organs, and intervene at infection sites. These processes are modulated by a complex array of chemokines that can be either constitutively expressed or induced upon inflammation [1, 2]. With few exceptions, most chemokines are thus considered to be either homeostatic or inducible [3]. Here, we examined whether high‐mobility group box 1 protein (HMGB1), to date considered an inflammatory protein, also contributes to the trafficking of lymphocytes under steady‐state conditions.

HMGB1 is a chromatin‐associated protein expressed in almost all cell types [4, 5] that promotes the assembly of transcription factors on DNA. HMGB1 is also well known as an alarmin, or damage‐associated molecular pattern molecule, and, as such, it plays a crucial role during acute and chronic inflammation [6, 7]. HMGB1 can be secreted by myeloid cells upon pathogen‐associated molecular pattern, damage‐associated molecular pattern, and cytokine‐mediated activation, or passively released by necrotic cells. Once in the extracellular milieu, HMGB1 can either recruit or activate myeloid cells, depending on the mechanism of its extracellular release, the receptor with which it may interact, and the presence of CXCL12. HMGB1 is known to interact with several immune cell receptors including TLR4, RAGE, TIM‐3, and CD24 [6]. Moreover, its pro‐inflammatory activity also depends on its binding partners: HMGB1 is often found in complexes with different molecules such as LPS, single‐strand DNA, and nucleosomes, and it can modulate their interaction with their respective receptor [8, 9]. The pro‐migratory and the activating functions of HMGB1 on myeloid cells are mutually exclusive, since they rely on different redox states of HMGB1 and on different receptors [10]. The disulfide form of HMGB1 can activate myeloid cells through the TLR4‐MD2 receptor complex in a NF‐kB dependent manner [11, 12], while the reduced or all‐thiol form can synergize and form a heterocomplex with the homeostatic chemokine CXCL12[13]. The HMGB1–CXCL12 complex induces different conformational changes in CXCR4 than CXCL12 alone, which results in an increased migration of fibroblasts and monocytes [13, 14, 15]. Thanks to its ability to both recruit and activate leukocytes, HMGB1 can thus play a crucial role in response to infections, as well as in the induction and regulation of trauma‐induced sterile inflammation [16].

Despite the well‐established multiple roles of HMGB1 on myeloid cells during innate immune responses, its impact on lymphoid cells is largely unknown. B lymphocytes are sensitive to CXCL12, which modulates their migration in different developmental stages and plays a critical role in regulation of tissue homeostasis, immune surveillance, and autoimmunity [17, 18]. Since HMGB1 is highly expressed throughout the organism, the HMGB1–CXCL12 complex could potentially influence B‐cell trafficking. In this work, we investigated the role of the HMGB1–CXCL12 complex on B‐lymphocyte migration during homeostasis. We show that the pro‐migratory role of the HMGB1–CXCL12 complex is not restricted to innate immune cells and fibroblasts but that it also applies to B lymphocytes. Moreover, the complex modulates B‐cell egress from Peyer's patches (PPs), showing a function of the HMGB1–CXCL12 complex under homeostatic conditions. The pro‐migratory function of the complex on B cells in vivo appears to be restricted to PPs, suggesting the existence of specific mechanisms regulating HMGB1–CXCL12 complex formation and/or function in different lymphoid organs. Importantly, the inhibition of the complex function decreases the intestinal IgA production, suggesting its potential as a target to modulate mucosal immune response.

## Results

### HMGB1 potentiates migration of B lymphocytes toward CXCL12

**Figure 1:**
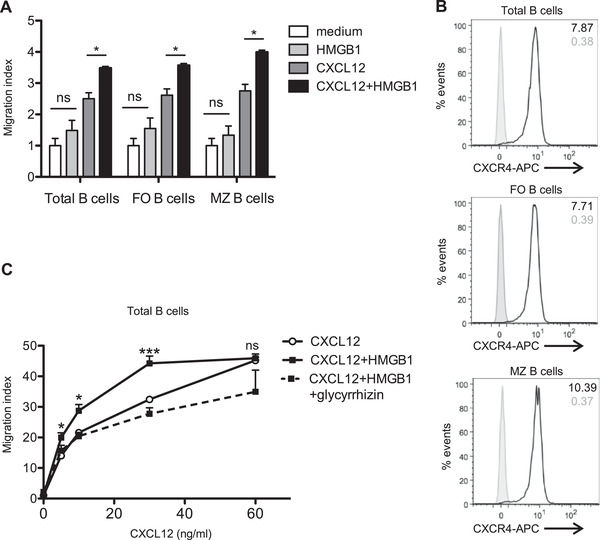
HMGB1 potentiates migration of B lymphocytes toward CXCL12. (A) Migration of splenic B lymphocytes toward 5 ng/mL CXCL12 ± HMGB1 300 nM, assessed using a modified Boyden chamber migration assay. Total, follicular (FO), and marginal zone (MZ) B cells are defined, respectively, as CD3‐B220^+^, CD3‐B220^+^CD21^+^CD23^+^, and CD3‐B220^+^CD21^high^CD23– cells. Pooled data of two independent experiments with one to two mice per experiment are shown (*n* = 2). All assays were done in technical triplicates. Means ± SEM are shown. Significance was measured with unpaired Student's *t*‐test. *p < 0.05. (B) CXCR4 expression measured by flow cytometry. Grey tinted histograms represent isotype controls. One representative experiment of three independent experiments is shown (*n* = 3). (C) Migration of total B lymphocytes toward different concentrations of CXCL12 ± 300 nM HMGB1 ± 200 μM glycyrrhizin. Pooled data of three independent experiments with one to two mice per experiment are shown (*n* = 3). All assays were done in technical triplicate. Significance was measured by two‐way ANOVA with Bonferroni post test correction. Mean ± SEM are shown. *p < 0.05, **p < 0.01.

To investigate whether HMGB1 enhanced lymphocyte recruitment toward CXCL12, we assessed migration of splenic B cells from naïve C57BL/6 mice toward HMGB1, CXCL12, or the combination of both in a transwell assay. HMGB1 increased the migration of total B cells and of the two main splenic B‐cell subsets, follicular and marginal zone B cells, in the presence of CXCL12 (Fig. 1A). In contrast, HMGB1 alone had no significant effect on B‐cell recruitment. Consistently, total B cells and both B‐cell subsets showed high expression of CXCR4, the receptor known to be essential for the promigratory function of the HMGB1–CXCL12 complex (Fig. 1B and Supporting Information Fig. S1).

We next performed a dose–response curve using different concentrations of CXCL12 to compare the number of migrated B cells in the presence or absence of HMGB1. The synergistic effect of HMGB1 on CXCL12‐induced B‐cell migration was most effective at suboptimal CXCL12 concentrations (Fig. 1C), as shown previously for myeloid cells and fibroblasts [13]. To confirm the specificity of the HMGB1 effect, we added the HMGB1‐inhibitor glycyrrhizin, which blocks the pro‐migratory function of the complex [19]. In the presence of HMGB1 and glycyrrhizin, cell migration toward CXCL12 did not significantly differ compared to migration toward CXCL12 alone, which is consistent with the ability of glycyrrhizin to block the effect of the HMGB1–CXCL12 complex. In summary, we show that in the presence of HMGB1, the CXCL12‐induced migration of different B‐cell subsets is increased.

### HMGB1 inhibition leads to B‐cell retention in the PPs

**Figure 2:**
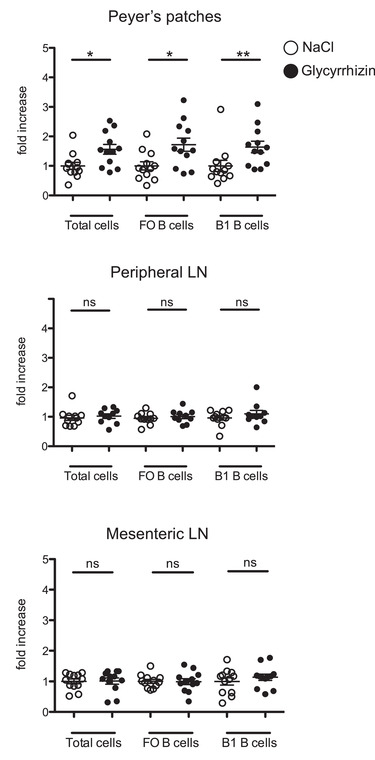
HMGB1 inhibition leads to B‐cell retention in the PPs. Number of total cells, FO B cells, and B1 B cells in PP, mesenteric LNs (MLNs), and peripheral LNs (PLN) in mice treated with NaCl (white dots) or glycyrrhizin (black dots). Mice were injected i.p. daily for 5 days with NaCl or 200 μg glycyrrhizin. At day 6, organs were harvested and cells were counted by flow cytometry. Results are expressed as fold increase compared to NaCl‐treated mice. FO B cells: CD3‐CD19^+^B220^+^CD21^+^CD23^+^ cells; B1 B cells: CD3‐CD19^+^B220^+^CD23‐CD5^+^ cells. Each dot represents one mouse (*n* = 12). Pooled data of three independent experiments are shown. Significance was measured by unpaired Student's *t*‐test. Means ± SEM are shown. *p < 0.05, **p > 0.01.

It is well described that CXCL12 can support the homing of naïve B lymphocytes to most secondary lymphoid organs, although its function is dispensable and secondary to CCL19 and CCL21 [20, 21]. In contrast, the CXCL12‐CXCR4 pathway has been reported to play a unique and essential role in promoting B‐cell egress specifically from PPs [22]. We postulated a role of the HMGB1–CXCL12 complex in B‐cell trafficking and hypothesized that HMGB1 inhibition would result in an increased number of B cells in the PPs. We treated mice with 200 μg glycyrrhizin i.p. for five consecutive days. This dosage was selected based on previous studies that tested the effects of different doses of glycyrrhizin on B‐cell functionality, in asthma and allergy mouse models [23, 24]. On day 6, we observed a higher number of total cells in the PP of glycyrrhizin‐treated mice compared to untreated mice (*p* = 0.012). This was due to an increase of both follicular (*p* = 0.012) and B1 (*p* = 0.0073) B‐cell subsets (Fig. 2). We did not observe any differences in numbers of T cells upon glycyrrhizin treatment (data not shown). In contrast, there was no difference in B‐cell numbers in either peripheral or mesenteric LNs, in accordance with the specific role of CXCL12 for promoting egress in the PPs. Our results suggest that the HMGB1–CXCL12 complex, rather than CXCL12 alone, is responsible for B‐cell exit from the PPs.

### Glycyrrhizin blocks egress from PPs only of CXCR4‐competent B cells

**Figure 3:**
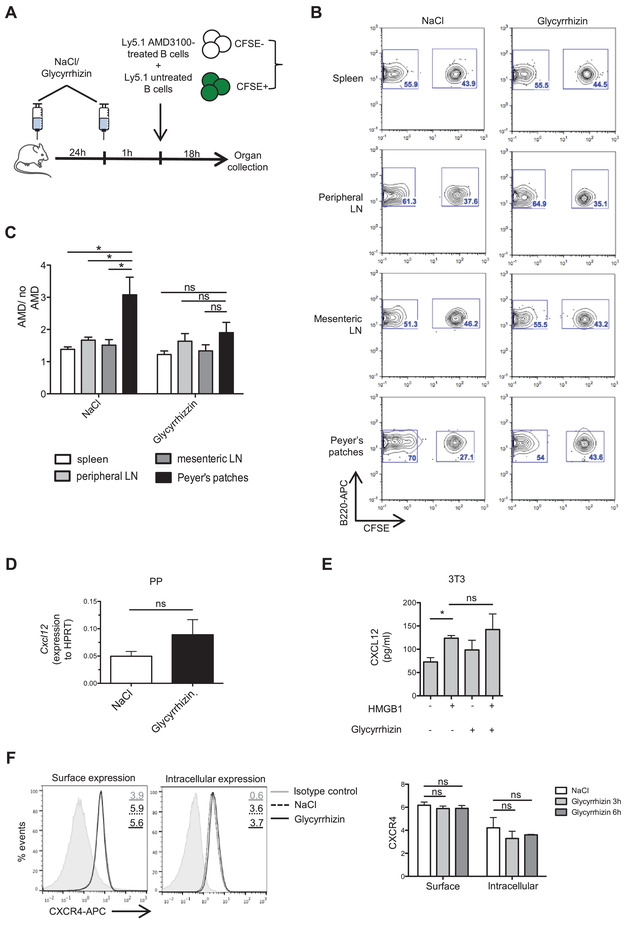
Glycyrrhizin inhibits egress from PPs of CXCR4‐competent B cells only. (A) Recipient C57BL/6 mice were injected twice at a 24‐h interval with NaCl or 200 μg glycyrrhizin. One hour after the second injection, equal proportions of AMD3100‐pretreated and CFSE‐stained, untreated B cells from congenic Ly5.1 mice were injected i.v. Lymphoid organs were collected and analyzed by flow cytometry 18 h after the transfer. (B) Representative dot plots for one mouse per group, gated on B220^+^CD45.1^+^ transferred cells, show the percentages of AMD3100‐pretreated (CFSE–) and untreated (CFSE^+^) cells in the different lymphoid organs. (C) Ratio of AMD3100‐pretreated/untreated cells for each organ. Means ± SEM of individual mice (*n* = 6) pooled from two independent experiments are shown. Significance was measured by unpaired Student's *t*‐test. *p < 0.05. (D) *Cxcl12* mRNA expression in PPs 4 h after glycyrrhizin injection and measured by qPCR. Data were normalized based on *Hprt* gene expression. Means ± SEM of individual mice (*n* = 4) pooled from two independent experiments are shown. Significance was measured by unpaired Student's *t*‐test. (E) CXCL12 protein levels in supernatant of 3T3 cells after overnight incubation with 300 nM HMGB1 ± 200 μM glycyrrhizin, measured by ELISA. Mean ± SEM of two independent experiments is shown. Significance was measured by unpaired Student's *t*‐test. *p < 0.05. (F) CXCR4 surface and intracellular expression on B cells from spleen and other secondary lymphoid organs measured by flow cytometry after incubation with 200 μM glycyrrhizin for 3 or 6 h. Representative histograms gated on CD3‐B220^+^ cells from individual mice stimulated for 6 h with glycyrrhizin (left) and mean ± SEM of mean fluorescence intensity (MFI) of three biological replicates (*n* = 3 mice) (right) are shown (pooled data of three independent experiment, with one mouse per experiment). Significance was measured by unpaired Student's *t*‐test. ns: nonsignificant.

To confirm that the glycyrrhizin‐mediated inhibition of B‐cell egress from the PPs depends on CXCR4, we performed an adoptive transfer experiment with B cells pretreated with AMD3100, a specific CXCR4 inhibitor [25]. Unlabeled AMD3100‐pretreated B cells and CFSE‐labeled, untreated B cells from C57BL/6 Ly5.1 mice were adoptively transferred in an equal ratio into congenic C57BL/6 mice that had been previously injected with glycyrrhizin or with NaCl (Fig. 3A). Eighteen hours later, we compared the ratio of AMD3100‐treated and untreated transferred B cells in PPs and in other secondary lymphoid organs by flow cytometry.

We observed no significant differences in the ratio of AMD3100‐pretreated and untreated transferred B cells in spleen, PLN, and MLN of either NaCl or glycyrrhizin‐injected mice. In accordance with the role of CXCR4‐CXCL12 pathway in promoting B‐cell egress from PPs [22], the adoptively transferred B cells pretreated with AMD3100 specifically accumulated in the PPs in mice injected with NaCl. On the contrary, in mice preinjected with glycyrrhizin, the ratio of AMD3100‐treated and untreated B cells was similar in PPs and in other lymphoid organs (Fig. 3B and C). This suggests that glycyrrhizin inhibits the egress of B cells from the PPs in a CXCR4‐dependent manner, since its main effect is on CXCR4‐competent B cells. Using an alternative labeling system, we confirmed that the increased ratio of AMD3100‐treated cells in the PPs is not due to a selective loss of CFSE‐labeled cells, and that AMD3100 pre‐treatment indeed induces an accumulation of cells specifically in the PPs independently of the labeling method (Supporting Information Figs. S2A and B). Of note, we confirmed in vitro that cells treated with AMD3100 still showed an impaired migration toward CXCL12 18 h after treatment (Supporting Information Fig. S7).

Our results suggest that glycyrrhizin delays the egress from PPs only of CXCR4‐functional B cells, in accordance with observations that the promigratory effect of the HMGB1‐CXCL12 acts via CXCR4 [13].

As it has previously been reported that HMGB1 can induce CXCL12 expression in fibroblasts via interaction with RAGE [13], we examined whether glycyrrhizin injection affected CXCL12 levels in mice. We did not detect any decrease in CXCL12 mRNA expression in PPs after treatment of mice with glycyrrhizin (Fig. 3D). Furthermore, glycyrrhizin did not decrease HMGB1‐induced CXCL12 production from 3T3 fibroblasts (Fig. 3E), suggesting that the HMGB1–RAGE interaction is not abolished by glycyrrhizin [19]. Moreover, the reduced CXCL12‐dependent B‐cell egress upon glycyrrhizin treatment was not due to a decreased surface or intracellular expression of CXCR4 in B cells (Fig. 3F). Taken together, these observations indicate that HMGB1–CXCL12 inhibition reduces the egress of B cells from PPs in a CXCR4‐dependent manner, without affecting either CXCR4 or CXCL12 expression.

### The HMGB1–CXCL12 complex does not play a role in germinal center polarization nor B‐cell egress from bone marrow

To examine whether the HMGB1–CXCL12 complex plays a role in other physiological situations besides lymphocyte egress from the PPs, we investigated the effect of the inhibition of the complex in two other of the most prominent CXCL12/CXCR4‐dependent B‐cell migratory mechanisms. The CXCL12‐CXCR4 pathway is known to play a critical role in the compartmentalization of germinal centers (GCs) that form in B‐cell follicles during a T‐cell‐dependent immune response. GCs are normally polarized into a dark and a light zone, containing centroblasts and centrocytes, respectively. In the absence of CXCR4, the polarization is lost [26]. In addition to centrocytes, the light zone also contains follicular DCs (FDCs) that can serve to discriminate the two zones. To assess the role of the complex in GC polarization, mice were immunized once with ovalbumin and treated daily with glycyrrhizin, AMD3100, or NaCl. Seven days after the immunization, the polarization of the newly generated GCs in the spleen was analyzed by immunofluorescence. In mice treated with NaCl or glycyrrhizin, there was no significant difference in the percentage of polarized GCs (Supporting Information Fig. S3A and B). In contrast, the AMD3100‐treated mice showed an FDC network extending throughout the GC and no clear distinction between dark and light zone in almost all GCs (>95%), as expected. Thus, the HMGB1–CXCL12 complex does not play a role in GC polarization in these conditions.

CXCL12 is highly expressed in the bone marrow where it serves as a retention signal for myelocytes. Treatment with AMD3100, which blocks CXCR4, causes the mobilization from the bone marrow of hematopoietic stem cells and immature B cells [27, 28, 29]. To examine whether the inhibition of the HMGB1–CXCL12 complex would similarly promote B‐cell mobilization, we treated mice with glycyrrhizin, AMD3100, or NaCl. Three hours later, we monitored by flow cytometry the percentage of immature B cells in the circulation [28]. Although AMD3100 treatment induced a clear increase in the percentage of immature IgD‐IgM‐ B cells in the blood without affecting the percentage of B cells in more advanced developmental stages (IgM^+^IgD–, IgM^+^IgDlow, and IgD^+^ B cells), glycyrrhizin did not modify circulating B‐cell populations (Supporting Information Fig. S3C and D), even at a later time point of 24 h (data not shown). Taken together, these results indicate that the HMGB1–CXCL12 complex does not play a role in major CXCL12‐dependent B‐cell mechanisms other than egress from PPs.

### PPs have more extracellular HMGB1 than other lymphoid organs

**Figure 4:**
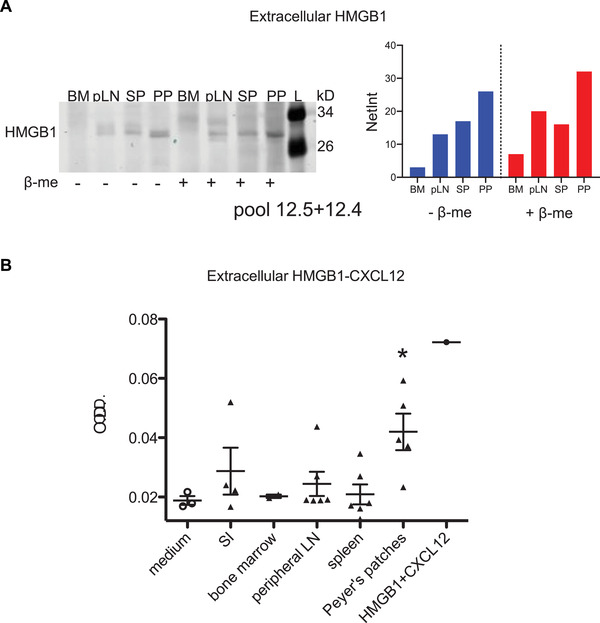
PPs have more extracellular HMGB1 than other lymphoid organs. (A) Extracellular HMGB1 in Peyer's patches (PP), spleen (SP), peripheral LNs (pLN), and bone marrow (BM), in reducing and nonreducing conditions, measured by immunoblot analysis. Quantification (right) has been obtained measuring band intensities, after background subtraction (Net Intensity, NetInt). Data are representative of three independents experiments. Bar graphs show the quantification of one representative experiment. Original blot picture in Supporting Information Fig. 4b. (B) Extracellular HMGB1–CXCL12 complex detected by hybrid ELISA in different lymphoid compartments and small intestine (SI). Results are expressed as absorbance at 450 nm after subtraction of absorbance at 570 nm. Mean ± SEM of individual mice (*n* = 3–6) is shown. Data are representative of at least five independent experiments. Significance was measured by one‐way ANOVA with Dunnet's posttest correction. *p < 0.05.

In view of the impact of the HMGB1–CXCL12 complex for lymphocyte migration in the PPs compared to other lymphoid organs, we assessed the amount of extracellular HMGB1 released in these organs. Bone marrow, peripheral LNs, spleen and PPs from naïve mice were digested with collagenase, and the medium bathing the organs was analyzed by Western blot (Fig. 4A). We clearly observed higher levels of extracellular HMGB1 in the PPs compared to the other lymphoid organs in reducing and nonreducing conditions.

We then looked for the presence of the HMGB1‐CXCL12 complex, using a hybrid ELISA [13]. Interestingly, the only lymphoid organs where we could consistently detect the presence of the HMGB1–CXCL12 complex were the PPs (Fig. 4B). Of note, the HMGB1–CXL12 complex was undetectable also in mesenteric LNs, which represent, together with the PP, the main intestinal secondary lymphoid organs (Supporting Information Fig. S4). The difference was not due to increased cell death during the organ processing, since the number of dead cells was similar in the different organs, nor to a different amount of CXCL12, as all organs showed similar levels (data not shown). To exclude a potential contamination of epithelial cells in the PP tissue, we analyzed also small intestine fragments (SI in Fig. 4B), collected from regions without PP. We could not detect the complex in intestinal fragments without PP. Thus, among the lymphoid organs examined, PPs showed the highest levels of extracellular HMGB1 under homeostatic conditions and were the only ones in which we consistently detected the formation of HMGB1–CXCL12 complex.

### Inhibition of the HMGB1–CXCL12 complex decreases intestinal IgA production

**Figure 5:**
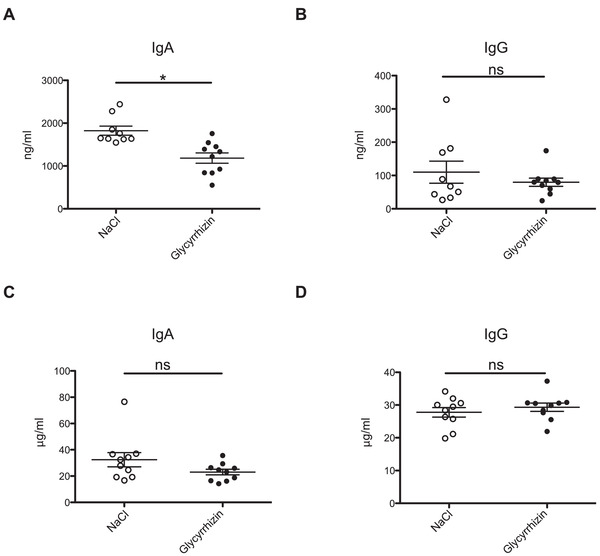
Glycyrrhizin decreases intestinal IgA production. IgA (A–C) and IgG (B–D) levels in the intestinal wash (A and B) and serum (C and D) of mice treated with physiological solution (white dots) and glycyrrhizin (black dots), measured by ELISA. Mice were injected i.p. daily for 5 days with NaCl or 200 μg glycyrrhizin. Eight hours after the last injection, mice were sacrificed and intestinal wash and blood were collected. Mean ± SEM of two independent experiments (*n* = 10 mice) is shown. Significance was measured by unpaired Student's *t*‐test. *p < 0.05.

PPs are the major inductive site for IgA‐producing B cells in the gut [30]. Once activated in the PPs, IgA^+^ effector B cells home to the intestinal lamina propria, where they terminally differentiate into IgA‐secreting plasma cells and release IgA antibodies in the intestinal lumen [31]. In order to evaluate the consequences of the HMGB1–CXCL12 complex inhibition in the gut, we measured the IgA intestinal content following glycyrrhizin treatment. We injected mice with 200 μg glycyrrhizin i.p. for five consecutive days, and collected the intestinal content 8 h after the last injection. Glycyrrhizin‐treated mice showed reduced intestinal IgA levels compared to untreated mice (Fig. 5A). In accordance with the specific role of PPs in regulating the gut IgA response, we did not observe any difference in the IgG intestinal content (Fig. 5B). In serum, we did not observe a significant difference for either IgA or IgG levels (Fig. 5C and D). Thus, HMGB1–CXCL12 complex inhibition is associated with a specific decrease in intestinal IgA production.

## Discussion

In this study, we have shown that HMGB1 enhances the recruitment of B lymphocytes toward CXCL12, unveiling the synergistic effect of HMGB1 and CXCL12 on B‐cell migration. We have moreover shown that HMGB1–CXCL12, acting through CXCR4, promotes B‐cell egress from the PPs under homeostatic conditions, and how its inhibition is associated with a reduced IgA intestinal production. HMGB1 has been previously reported to affect B cells through other receptors than CXCR4: it influences B‐cell activation in response to endogenous TLR9 ligands [32], and, in inflammatory bowel disease, an association between HMGB1 serum levels and IL‐8 production from B cells was observed, in a mechanism involving TLR2 and CD36 [33]. HMGB1 can thus modulate B‐cell functions in different ways, interacting with multiple receptors.

One of the major determinants of the extracellular functions of HMGB1 is its redox status. This status depends on the mechanism of HMGB1 release from the cell and on the redox conditions of the extracellular compartment; notably only fully reduced HMGB1 forms a complex with CXCL12 [10, 34]. Interestingly, we detected the HMGB1–CXCL12 complex only in PPs, suggesting organ‐specific environmental conditions. When HMGB1 is actively secreted by innate immune cells, a mix of the all‐thiol (fully reduced) and disulfide (oxidized) forms is usually present in the extracellular milieu [10]. We speculate that PPs may possess an anti‐oxidant microenvironment that favors the maintenance of HMGB1 in the reduced state. However, it is known that reducing conditions, which are necessary for efficient T‐cell activation during an adaptive immune response, are also present in LNs [35, 36] where we could not observe a potential effect of the heterocomplex; thus, it is unlikely that the existence of a reducing environment in the PPs is the sole determinant that justify the selective presence of the HMGB1–CXCL12 complex in the PPs.

Another major factor that determines the formation of the HMGB1–CXCL12 complex is local availability of binding partners. In contrast to the peripheral LNs, where CXCL12 is expressed mainly in the medullary cords [37], in PPs CXCL12‐producing cells are abundant in the interfollicular and subserosal areas, in close association with lymphatic sinuses [22] that represent the main egress route for lymphocytes. Although we did not definitely identify the source of extracellular HMGB1 in PPs, we observed cytoplasmatic HMGB1, which is indicative of active secretion [38], in CD11c^+^ cells located in the PP subserosal region (Supporting Information Fig. S5B). This observation suggests that subserosal CD11c^+^ DCs, which are in close proximity to CXCL12‐producing cells and lymphatic vessels, are a source of extracellular HMGB1. We speculate that the local release of both HMGB1 and CXCL12 allows the formation of the complex in the subserosal and interfollicular regions of PPs, and that this in turn facilitates the access of B lymphocytes to these areas, and consequently their egress from PPs.

Another possible explanation for the higher levels of the HMGB1–CXCL12 complex relies on the fact that PPs, unlike other secondary lymphoid organs, are chronically activated by microbiome‐ and food‐derived antigens and pathogen‐associated molecular patterns [39, 40]. The constant inflammatory conditions in the PP microenvironment may thus promote a higher release of HMGB1 from activated DCs [41] compared to other lymphoid organs, even under homeostatic conditions.

PPs play a crucial role in regulating the immune response toward intestinal pathogens and commensal microbiota. Indeed, the great majority of gut B cells are activated in the PPs, where they undergo the immunoglobulin class switch recombination from IgM to IgA. IgA^+^ B cells subsequently localize from the PPs to the lamina propria of the small intestine, where they terminally differentiate in IgA‐secreting plasma cells [31]. The decreased intestinal content in IgA that we observed upon glycyrrhizin treatment and the comparable serum levels of IgA support the hypothesis of a homeostatic role for extracellular HMGB1 in the gut, although our data are insufficient to claim that the observed effect is uniquely due to impaired B‐cell egress from PPs induced by HMGB1–CXCL12 complex blockade. Reduced IgA secretion into the intestinal lumen may dramatically affect the mucosal immune response by altering the microbiota composition and increasing susceptibility to mucosal viruses and bacterial infections. Our results suggest a novel role for the HMGB1–CXCL12 complex in the regulation of intestinal immune responses, possibly by tuning the egress of B cells from PPs and subsequently influencing luminal IgA antibody levels.

Of note, we did not detect any change in surface and intracellular expression of CXCR4 upon glycyrrhizin and glycyrrhizin alone does not influence CXCL12‐induced migration of B cells (Supporting Information Fig. S6). Our results indicate that glycyrrhizin alone does not influence the binding between CXCL12 and CXCR4, because this would cause the receptor internalization. This observation could be of importance to elucidate the mechanism of action of the HMGB1–CXCL12 heterocomplex on CXCR4, but further experiments are needed to achieve this goal.

We have described here a novel role for the HMGB1–CXCL12 complex in modulating B‐cell trafficking under homeostatic conditions, but it is possible that HMGB1 or HMGB1‐CXCL12 may exert further promigratory effects on lymphocytes during acute or chronic inflammation. Since CXCL12 is a crucial chemo‐attractant for B cells and modulates several pathological and physiological B‐cell processes, and HMGB1 is broadly expressed in almost all tissues, it is conceivable that the HMGB1–CXCL12 complex plays an as yet unrecognized role in other B‐cell migratory mechanisms that are currently considered to be mediated by CXCL12 alone, such as migration of plasma cells to the bone marrow or even leukemic cell dissemination [37, 42, 43, 44]. The observation that HMGB1 itself can induce CXCL12 production supports this hypothesis [13, 45]. Since physiological and pathological processes are commonly regulated by the concomitant expression of multiple factors, further studies will be necessary to characterize in detail the differential functions of chemokine heterocomplexes [46].

In conclusion, we demonstrate a homeostatic role for HMGB1, hitherto considered an inflammatory alarmin, and a pro‐migratory role of the HMGB1–CXCL12 complex on B lymphocytes in the PPs and on IgA antibodies production in the intestine. In view of the key function of PPs in the response to gut pathogens and commensal bacteria, this study provides a rationale to further investigate the role of the HMGB1–CXCL12 complex in regulating cell trafficking at mucosal sites, and describes a new mechanism that could be potentially targeted to modulate mucosal immune responses.

## Materials and Methods

### Mice and treatments

C57BL/6 and C57BL/6‐Ly5.1 mice were purchased from Janvier Labs (Le Genest‐Saint‐Isle, France). Mice were maintained in specific‐pathogen free conditions, and used between 6 and 14 weeks of age. All animal experimentation procedures were performed at the University of Fribourg (Switzerland), according to the Swiss federal legislation. In the indicated experiments, AMD3100 octahydrocloride (Sigma‐Aldrich, Sant Louis, MO) and glycyrrhizin (Minophagen Pharmaceutical, Tokyo, Japan) were injected i.p. at 3 mg/kg and 10 mg/kg, respectively, in 100 μL of 0.9% NaCl. For ovalbumin immunization, mice were injected i.p. with 10 μg EndoFit Ovalbumin in Alhydrogel adjuvant 2% (both from InVivogen, San Diego, CA). For adoptive cell transfer, single cell suspensions were obtained from spleen, peripheral, and mesenteric LNs and PPs of C57BL/6 Ly5.1 mice, and B cells were negatively isolated by Pan B‐cell isolation kit (Miltenyi Biotec, Bergisch Gladbach, Germany). Half of the cells were stained with 0.5 μM CFSE (Biolegend) following the manufacturer's protocol. The remaining cells were incubated with 500 μg/mL AMD3100 for 45 min at RT in RPMI medium without FCS. After washing, AMD3100‐treated and untreated cells were mixed in an equal ratio and 4 × 10^6^ cells were injected i.v. into congenic C57BL/6. Note that 18 h later the ratio of AMD3100‐treated and untreated cells in each organ was assessed and divided by the input ratio. For the analysis of mobilized cells from the bone marrow, blood was obtained 3 and 24 h after the indicated treatments.

### Chemotaxis assay

Chemotaxis was assessed by a modified Boyden chamber migration assay using a microplate with a 3 μm pore filter (Neuro Probe, Gaithersburg, MD). After red cell lysis (BD Pharm Lyse; BD Biosciences), freshly isolated splenocytes were rested in RPMI 1640 with 10% FCS (MP Biomedicals, Santa Ana, CA), 1% sodium pyruvate, 5 × 10^–5^ M 2‐mercaptoethanol, 1% HEPES, 2 mmol/L l‐glutamine (PAA LABORATORIES, Pasching, Austria), 50 U/mL penicillin, 50 μg/mL streptomycin (Corning, Manassas, VA), for at least 4 h at 37°C prior to the assay. LPS‐free fully reduced HMGB1 (HMGBiotech s.r.l., Milan, Italy), CXCL12 (PeproTech, London, UK), and glycyrrhizin were diluted in the same medium at the indicated concentration, and added to the bottom chamber of the chemotaxis plate. HMGB1 was used at the concentration of 300 nM, as described [13]. Total splenocytes (5 × 10^5^ per well) were added in the top wells and allowed to migrate for 3 h. The migrated cells were recovered from the bottom wells, stained with propidium iodide, and B lymphocytes were counted by flow cytometry. The migration index was calculated by dividing the number of migrated cells in each well by the number of those migrated toward medium alone.

### Flow cytometry

Single cell suspensions were obtained from lymphoid organs by gently passing through a 40 μM cell strainer in PBS containing 0.5% BSA and 0.5 mM EDTA. Cells were incubated for 10 min with Fc receptor‐blocking antibody (TruStain fcX; Biolegend, San Diego, CA) and stained with the following fluorescently labeled antibodies: anti‐mouse B220 (RA3‐6B2), CD3 (17A2), CD45.1 (A20), CD19 (6D5), CD21/CD35 (7E9), CD23 (B3B4), IgM (RMM‐1), IgD (11‐26c.2a), CD5 (53‐7.3), all from Biolegend, and CXCR4 (2B11) from eBioscience (San Diego, CA). Alexafluor 647 Mouse IgG2b, k (MPC‐11; Biolegend) was used as isotype control. For CXCR4 intracellular staining, cells were fixed with PFA 4% in PBS for 15 min at 4°C and permeabilized with saponin 0.2% in PBS containing 0.5 mM EDTA and 0.5% BSA. Acquisition was performed on a MACSQuant Analyzer (Milteny Biotec) and data analysis was done using FlowJo vX.0.7 software (Treestar, Ashland, OR). All flow cytometry experiments were performed adhering to the “Guidelines for the use of flow cytometry and cell sorting in immunological studies” [47].

### ELISA

The concentration of CXCL12 in 3T3 cells and tissue supernatant was evaluated with DuoSet ELISA kit (R&D Systems, Minneapolis, MN), using 10^6^ 3T3 cells per well. For the analysis of intestinal IgA and IgG content, intestine was flushed with PBS containing 0.05 M EDTA and 66 μM PMSF; the fluid was recovered and centrifuged, and the supernatant applied on the ELISA plate, after 1:1 dilution in 0.5% BSA solution. A goat anti‐mouse IgA or anti‐mouse IgG (SouthernBiotech Birmingham, AL) were used as coating antibodies, and a goat anti‐mouse IgA (alpha‐chain specific) or anti‐mouse IgG (gamma chain specific) antibodies coupled to peroxidase (Sigma‐Aldrich, Sant Louis, MO) were used as detection antibodies. For the hybrid ELISA for HMGB1‐CXCL12 complex detection, organs were mechanically digested in PBS with a syringe plunger and passed through a 40 μM cell strainer. For bone marrow tissue, tibia and femurs were flushed with PBS and processed as the other organs. Intact cells were removed by centrifugation at 400 g for 30 min, and the supernatant was collected. The supernatant was concentrated 10× using 3K Microsep Advance Centrifugal Device (Pall Corporation, Ann Arbor, MI) following the manufacturer's protocol, and applied on the ELISA plate. The hybrid ELISA was then performed as described previously [13]. As positive control, all‐thiol‐HMGB1 (0.1 μg/mL) was preincubated with CXCL12 (0.1 μg/mL) at 37°C for 15 min, before performing the assay. As negative control, sample buffer was used.

### Immunoblot analysis

To analyze extracellular HMGB1, organs were sized to obtain the same amount of initial tissue and incubated in PBS containing 3 mg/mL of collagenase type IV (Worthington, Lakewood, NJ) and 1× Halt proteases inhibitor cocktail (Thermo Fisher Scientific, Waltham, MA), for 30 min. The medium was diluted in sample buffer (Tris‐HCl [pH 6.8], 6% SDS and 30% glycerol) and supplemented with 14.1 M β‐mercaptoethanol for reducing conditions, boiled for 5 min at 95°C and loaded in 12% acrylamide gels, followed by electrophoresis and transfer onto nitrocellulose membranes (GE Healthcare, Buckinghamshire, UK). Membranes were blocked with skim milk and rabbit polyclonal anti‐HMGB1 antibody was applied overnight. Anti‐rabbit Dylight 680 (Cell Signaling, Dancers, MA) was used as secondary antibody, and membranes were analyzed with a LiCor fluorescence reader (LiCor, Lincoln, NE).

### Quantitative real‐time PCR

PP tissue was mechanically disrupted and homogenized in Buffer RLT Plus (Qiagen, Helden, Germany), and total RNA was extracted with RNeasy Plus Mini kit (Qiagen), following manufacturer's instructions. A total of 1 μg of RNA per sample was used to obtain cDNA, and the qPCR were performed as described [48]. The following primers were used: Cxcl12: 5′‐TATAGACGTGGCTTTGCAG‐3′ (forward) and 5′‐TTGACTCAGGACAAGGCATC‐3′ (reverse); Hprt: 5′‐ATGAGCGCAAGTTGAATCTG‐3′ (forward) and 5′‐CAGATGGCCACAGGACTAGA‐3′ (reverse).

### Immunofluorescence

Fresh tissues were embedded in O.C.T mounting media (VWR international, Dublin, Ireland) and frozen in an isopentane bath in liquid nitrogen. Note that 5–7 μm sections were prepared using a Leica CM1860 UV cryostat (Leica Biosystems, Wetzlar, Germany). Sections were fixed in cold acetone or PFA 4% in PBS for 15 min, and unspecific antibody binding was blocked using BSA 3% in PBS for 30 min. Sections were incubated overnight at 4°C with the primary antibody, and 2 h at RT with the secondary. The cell nuclei were counterstained with DAPI. The following primary antibodies were used: rat anti‐mouse FDC‐M2 (FDC‐M2; Amsbio, Abingdon, UK), rat anti‐mouse IgD (11‐26c.2a; Biolegend), rabbit anti‐mouse Ki‐67 (SP6; Abcam, Cambridge, UK), rabbit polyclonal anti‐HMGB1 (Abcam), and armenian hamster CD11c (N418; Biolegend). As secondary antibodies were used: donkey anti‐rabbit Alexa Fluor 488, goat anti‐armenian hamster Alexa Fluor 488 (Jackson ImmunoResearch, West Grove, PA), goat anti‐rat Alexa Fluor 555, and a goat anti‐rabbit Alexa Fluor 568 (Life technologies, Carlsbad, CA). Pictures were taken with a Nikon Eclipse Ni‐U microscope (Nikon Instruments, Amsterdam, The Netherlands). For the analysis of GC polarization, slides were scanned with a Hamamatsu Nanozoomer (Hamamatsu Photonics, Japan). Several cross‐sections for each spleen, spanning at least 150 μM in depth, were stained and the numbers of total and polarized GC were counted. The mean for all cross‐sections was calculated for each mouse.

### Statistical analysis

Statistical significance was evaluated using two‐tailed Student's *t*‐test and one‐ or two‐way ANOVA with Dunnett or Bonferroni posttest correction, when appropriate. *p*‐Values < 0.05 were considered significant. Statistical analysis was done with GraphPad Prism 5 software (GraphPad Softwares Inc., CA).

## Conflict of interest

The authors declare that they have no commercial or financial conflict of interest. However, M.E.B. is founder and part‐owner of HMGBiotech, a company that provides goods and services related to HMGB proteins.

## Author contributions

L.S., C.B., and M.U. conceived the study. L.S., A.O., T.S., N.B., and J.W. performed experiments and analyzed data. L.S., V.P., and C.B. wrote the manuscript. M.U., M.E.B., I.M., and C.H. discussed the results and edited the manuscript.

### Peer review

The peer review history for this article is available at https://publons.com/publon/10.1002/eji.202049120.

**Abbreviations**
FDC: follicular DC
HMGB1: high‐mobility group box 1 protein
PP: Peyer's patch

## Supporting information



Supporting Information

## Data Availability

The data that support the findings of this study are available from the corresponding author upon reasonable request.
