# Supplementary material for: HMGB1 promotes CXCL12‐dependent egress of murine B cells from Peyer's patches in homeostasis
Source: Eur J Immunol. 2021 Jun 16;51(8):1980–91. doi: 10.1002/eji.202049120 (PMC8453951; doi:10.1002/eji.202049120)
Supplement: Supplementary file 1 — Supporting Information [file EJI-51-1980-s001.pdf]

Supporting information

Supplementary figure legends

Figure S1: Follicular and marginal zone B cells in spleen show similar CXCR4 surface expression.

CXCR4 expression measured by flow cytometry on follicular (FO) and marginal zone (MZ) B cells (blue and red lines, respectively), freshly isolated (dotted lines) and after overnight incubation in CXCL12-free medium (solid lines). As negative control, isotype antibody (light grey) and CXCR4 expression on freshly isolated T lymphocytes (CD3+B220- cells) are shown (dark grey). The histograms representing FO and MZ B cells (o.n.) are the same shown in main figure 1b.

Figure S2: Increase percentage of AMD3100-treated cells in PPs is not due to a selective loss of CFSE-labeled cells.

A) Naïve mice were adoptively transferred with an equal amount of CFSE and e670-labeled cells through i.v. injection. Lymphoid organs were collected and analyzed by flow cytometry 18 h after the transfer, and the ratio of the differentially labeled cells were calculated as in Figure 3. Each dot represents one mouse (n=2). B) e670-labeled cells were treated with AMD3100 as in Figure 3, while CFSE-labeled cells were left untreated, and the differentially labeled cells were adoptively transferred in equal amounts into naïve mice. Each dot represents one mouse (n=5). Significance was measured by unpaired Student t-test. *$p<0.05$, **$p<0.01$.

Figure S3: HMGB1-CXCL12 does not play a role in germinal center polarization or in B cell egress from bone marrow.

A) Mice were immunized with OVA and treated daily for 6 d with NaCl, AMD3100 or glycyrrhizin. Spleens were collected on day 7. Cross-sectional view of GC in the spleen of one representative mouse per group, stained for Ki-67 to visualize GC B cells (green), FDC-M2 for follicular dendritic cells (red) and DAPI (blue). Scale bar is 100 μM. B) Ratio of polarized GC/total GC. Mean ± SEM of individual mice is shown (n=2). Data are representative of two independent experiments. C and D) Mice were injected once i.p. with NaCl, AMD3100 or glycyrrhizin and circulating B cells were analyzed by flow cytometry 3 h later. C) Dot plots of one representative mouse per group, gated on CD19+ cells and stained for IgD and IgM, are shown. D) Percentages of subpopulations within CD19+ B cells. Mean ± SEM of individual mice is shown (n=4). Representative data of at least two independent experiments are shown. Significance was measured by unpaired Student t-test. *$p<0.05$.

Figure S4: PPs present a higher amount of extracellular HMGB1-CXCL12 complex than mesenteric lymph nodes.

A) HMGB1-CXCL12 complex detected by hybrid ELISA as in figure 4b. Results are expressed as absorbance at 450 nm after subtraction of absorbance at 570nm, normalized with respect to the number of cells in the starting tissue. Mean± SEM of individual mice (n=3) is shown.

B) Original uncropped blot image shown in main Figure 4a. The black arrow indicates the HMGB1 bands. In the main figure is shown the mirror image of the cropped area containing HMGB1 bands.

Figure S5: Subserosal CD11c+ cells present cytoplasmatic HGMB1 in PPs.

A) Representative fluorescent microscopy image of a PP cross-section stained for CD11c (green), IgD for naïve B lymphocytes (red) and DAPI (blue). B) PP section stained for CD11c (green),

HMGB1 (red) and DAPI (blue). Arrows in the high-magnification images indicates CD11c+ cells with cytoplasmatic HMGB1 expression. The dotted white line marks the outer border of the PP. Scale bars, 100μM.

Figure S6: Glycyrrhizin alone does not influence CXCL12-induced cell migration. Migration of splenocytes towards 100ng/ml CXCL12 ± 200μM glycyrrhizin, assessed using a modified Boyden chamber migration assay. Mean± SEM are shown. Significance was measured with unpaired Student's t test.

Figure S7: AMD3100-treated cells showed impaired migration towards CXCL12 18h after treatment.

Migration of untreated and AMD3100-treated splenocytes towards 100ng/ml CXCL12, assessed using a modified Boyden chamber migration assay. Cells were treated or not with 10μg/ml AMD3100 for 30min, washed and re-suspended in drug-free medium. 18h later, cell viability was checked by propidium iodide staining, and the migration assay was performed as described in Figure 1. For each group, the migration index was calculated by dividing the number of migrated cells in each well by the number of those migrated toward medium alone. Mean ± SEM of duplicates is shown. Significance was measured by Student t-test. **$p<0.01$.

Figure S8: Gating strategies of flow cytometry experiments.

A) Gating strategy relative to Figure 2 identifying follicular B cells (FO B cells, CD3-CD19+B220+CD21+CD23+) and B1 B cells (CD3-CD19+B220+CD23-CD5+). B) Gating

strategy relative to Figure 3 identifying adoptively transferred B cells from Ly5.1 mice, CFSE-labelled (B220+CD45.1+CFSE+) and unlabeled (B220+CD45.1+CFSE-).

Supplementary figures

Figure S1

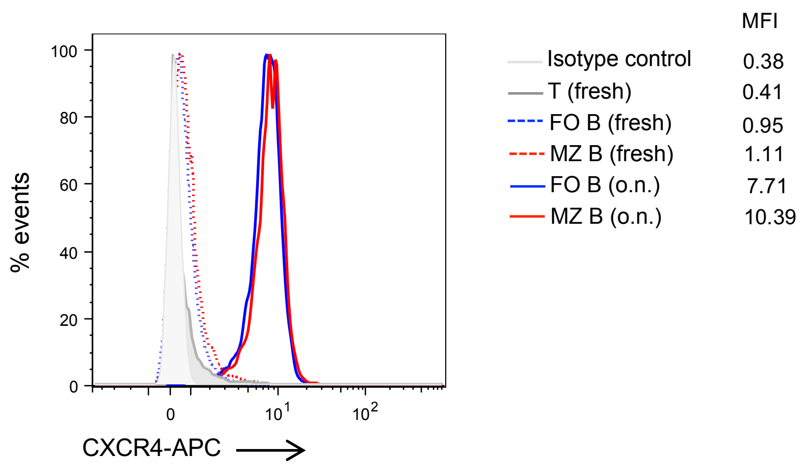

Figure S2

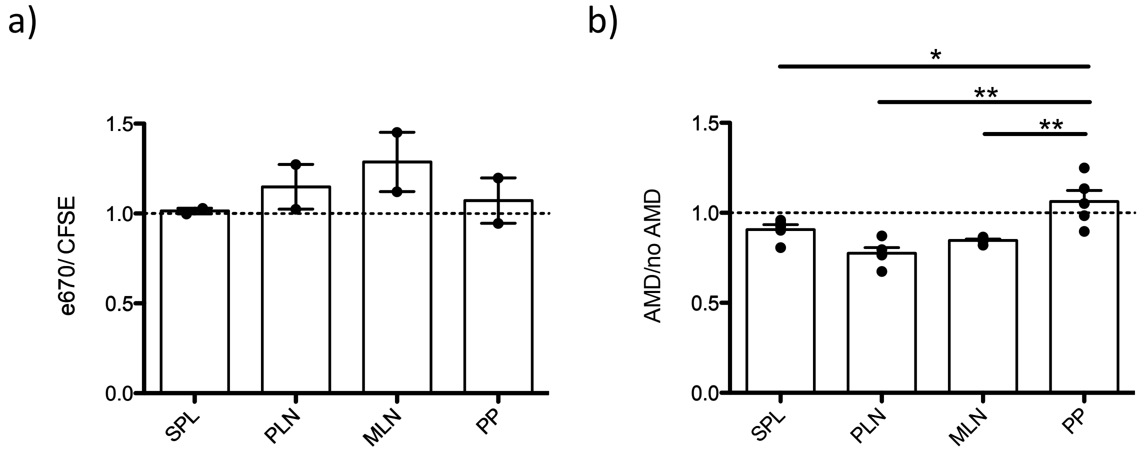
a)
e670/ CFSE
1.5
1.0
0.5
0.0
SPL
PLN
MLN
PP
b)
AMD/no AMD
*
**
**
1.5
1.0
0.5
0.0
SPL
PLN
MLN
PP

Figure S3

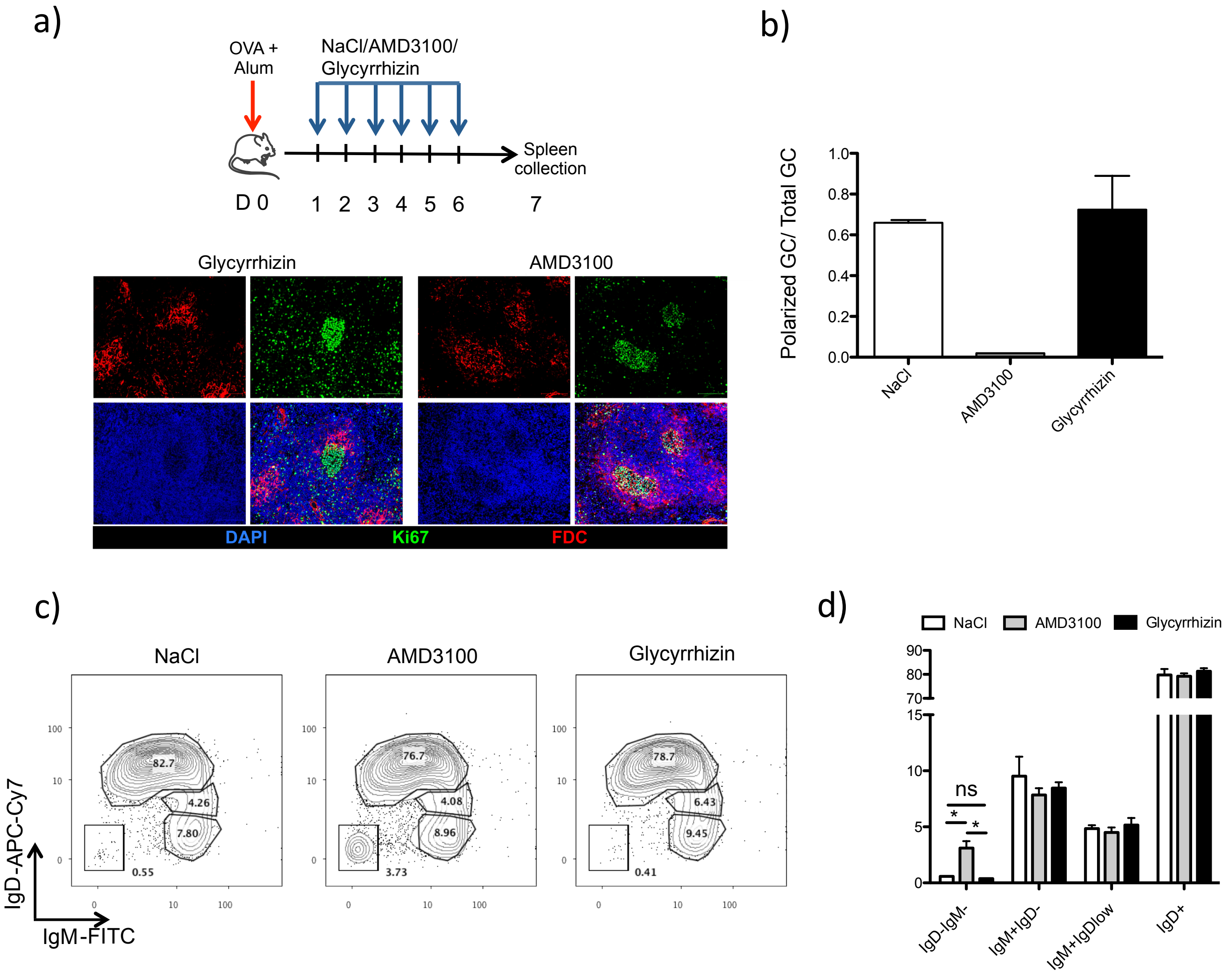

a)
OVA + Alum
NaCl/AMD3100/ Glycyrrhizin
Spleen collection
D 0
1 2 3 4 5 6
7
Glycyrrhizin
AMD3100
DAPI
Ki67
FDC
b)
Polarized GC/ Total GC
1.0
0.8
0.6
0.4
0.2
0.0
NaCl
AMD3100
Glycyrrhizin
c)
NaCl
AMD3100
Glycyrrhizin
IgD-APC-Cy7
IgM-FITC
82.7
4.26
7.80
0.55
76.7
4.08
8.96
3.73
78.7
6.43
9.45
0.41
d)
NaCl
AMD3100
Glycyrrhizin
90
80
70
15
10
5
0
ns
*
*
IgD-IgM-
IgM+IgD-
IgM+IgDlow
IgD+

Figure S4

a)

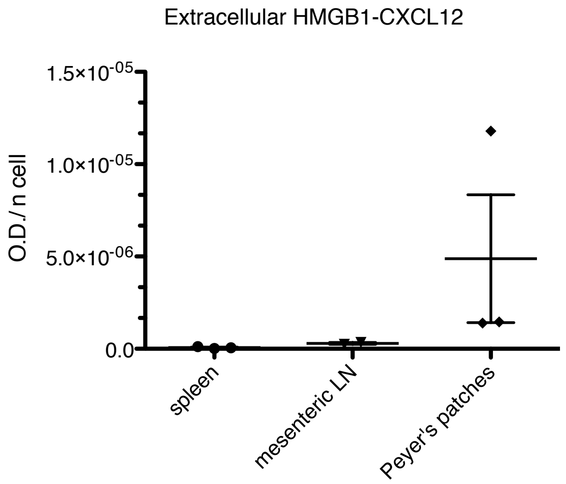
Extracellular HMGB1-CXCL12
O.D./ n cell
1.5×10-05
1.0×10-05
5.0×10-06
0.0
spleen
mesenteric LN
Peyer's patches

b)

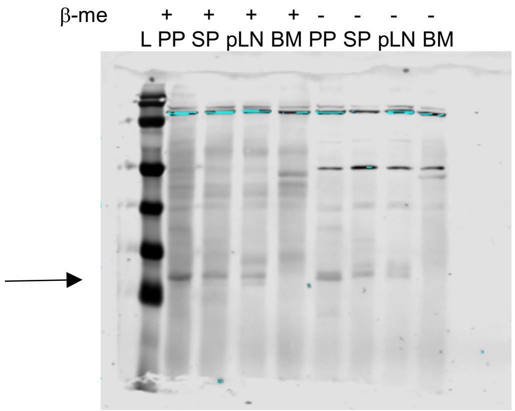
β-me
+ + + + - - - -
L PP SP pLN BM PP SP pLN BM

Figure S5

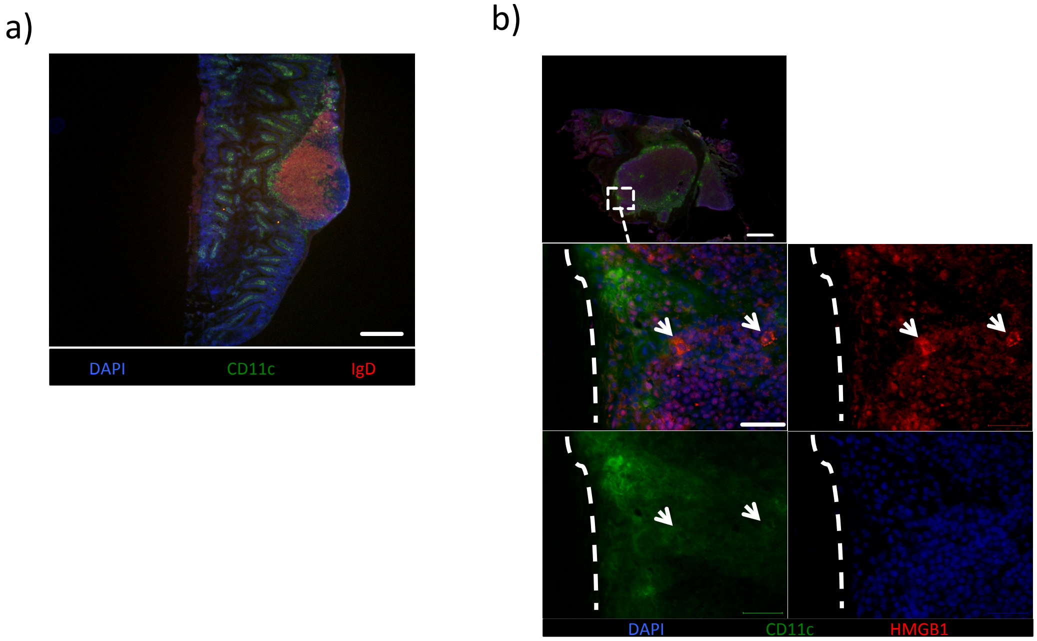

Figure S6

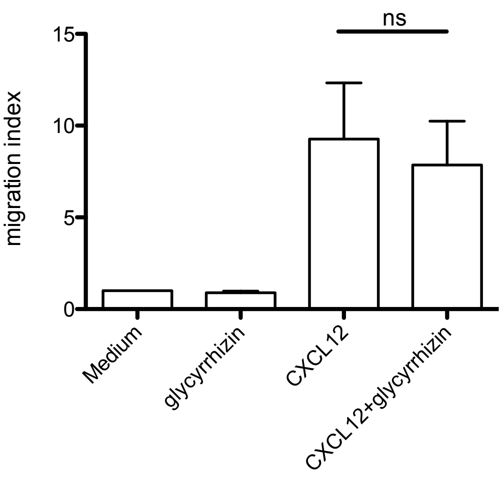
ns
15
10
5
0
migration index
Medium
glycyrrhizin
CXCL12
CXCL12+glycyrrhizin

Figure S7

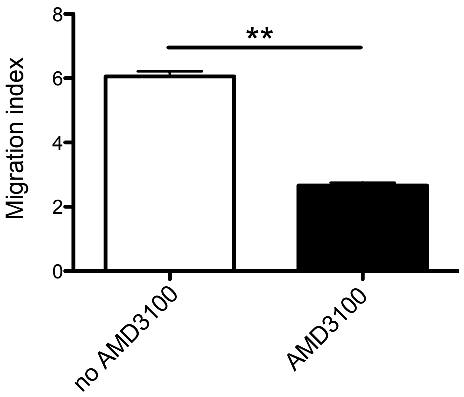

Figure S8

a)

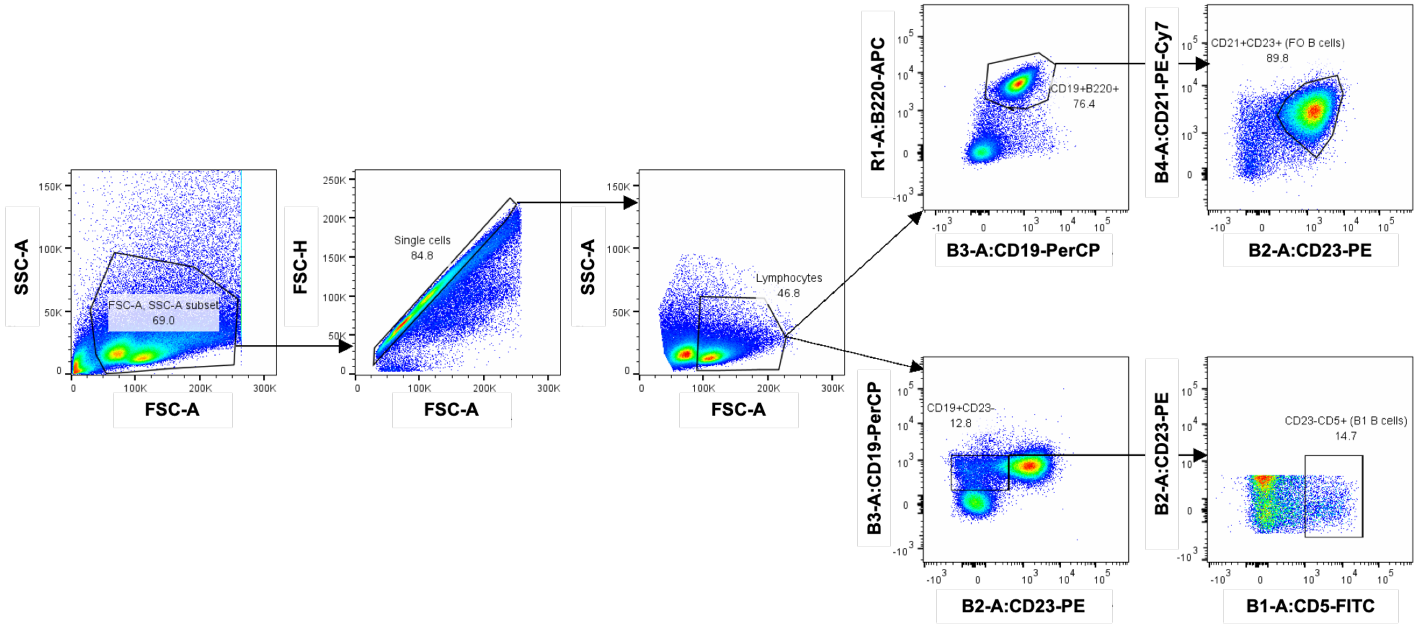

SSC-A
FSC-A
FSC-A, SSC-A subset
69.0
FSC-H
Single cells
84.8
Lymphocytes
46.8
R1-A:B220-APC
B3-A:CD19-PerCP
CD19+B220+
76.4
B4-A:CD21-PE-Cy7
B2-A:CD23-PE
CD21+CD23+ (FO B cells)
89.8
B3-A:CD19-PerCP
CD19+CD23-
12.8
B2-A:CD23-PE
B1-A:CD5-FITC
CD23-CD5+ (B1 B cells)
14.7

b)

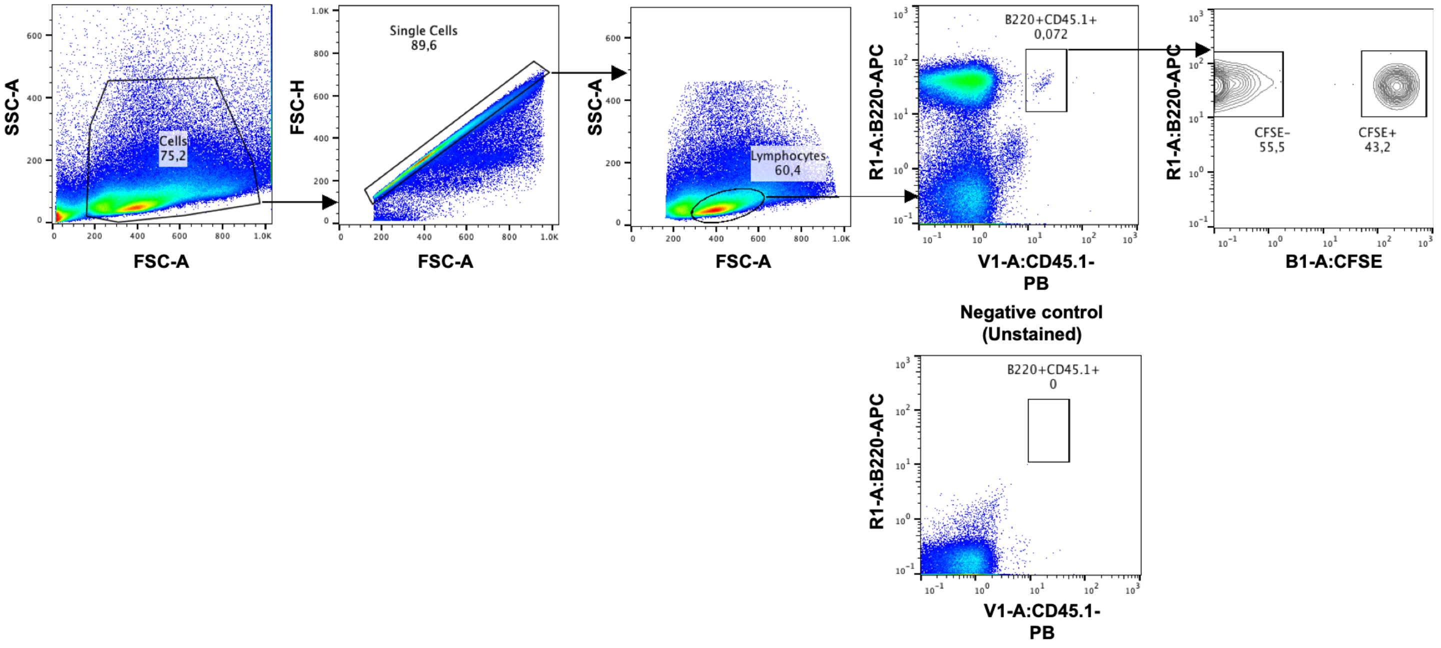

SSC-A
FSC-A
Cells
75,2
FSC-H
Single Cells
89,6
Lymphocytes
60,4
R1-A:B220-APC
V1-A:CD45.1-PB
B220+CD45.1+
0,072
B1-A:CFSE
CFSE-
55,5
CFSE+
43,2
Negative control (Unstained)
B220+CD45.1+
0
